# *Mycobacterium tuberculosis* Dissemination Plays a Critical Role in Pathogenesis

**DOI:** 10.3389/fcimb.2020.00065

**Published:** 2020-02-25

**Authors:** Madeleine G. Moule, Jeffrey D. Cirillo

**Affiliations:** Department of Microbial Pathogenesis and Immunology, Texas A&M University Health Science Center, Bryan, TX, United States

**Keywords:** tuberculosis, *Mycobacterium*, dissemination, extrapulmonary, pathogenesis

## Abstract

*Mycobacterium tuberculosis* is primarily a respiratory pathogen. However, 15% of infections worldwide occur at extrapulmonary sites causing additional complications for diagnosis and treatment of the disease. In addition, dissemination of *M. tuberculosis* out of the lungs is thought to be more than just a rare event leading to extrapulmonary tuberculosis, but rather a prerequisite step that occurs during all infections, producing secondary lesions that can become latent or productive. In this review we will cover the clinical range of extrapulmonary infections and the process of dissemination including evidence from both historical medical literature and animal experiments for dissemination and subsequent reseeding of the lungs through the lymphatic and circulatory systems. While the mechanisms of *M. tuberculosis* dissemination are not fully understood, we will discuss the various models that have been proposed to address how this process may occur and summarize the bacterial virulence factors that facilitate *M. tuberculosis* dissemination.

## Introduction

Tuberculosis is one of the oldest known human pathogens. The disease can be traced back through historical references and evidence of infections in human remains from some of the most ancient civilizations. Evidence of tuberculosis infections has been found in the necropoli of Ancient Egypt, Neolithic skeletons from burial sites in Europe, and mummies excavated from the Andes Mountains in South America (Formicola et al., 1987; Zink et al., 2001). The oldest confirmed human tuberculosis patient dates back an estimated 9,000 years ago, from submerged site in the Mediterranean near modern day Israel, but statistical models have estimated that the *Mycobacterium tuberculosis* complex may have evolved 40,000 years ago around the same time that human populations are thought to have begun to expand and migrate out of Africa (Hershkovitz et al., 2008; Wirth et al., 2008).

This bacterial pathogen has followed and affected humans throughout history, and has become an infection so familiar that it has taken root in our collective understanding of health and disease. References to a disease thought to be tuberculosis can be found in the Torah and the Old Testament of the Bible, and in written documents from China and India that are over 200 years old. Descriptions made by Hippocrates in Ancient Greece indicate that physicians and scientists have been attempting to study tuberculosis for as long as the practice of medicine has existed (Barberis et al., 2017). In more recent times the extremely high incidence of tuberculosis, or “consumption,” in Europe and the Americas led to an extended campaign against what was termed the “White Plague,” resulting in establishment of sanatoriums that were so ubiquitous that they are still commonly referenced in the literature and other media today (Martini et al., 2018).

Despite the long history of attempts to understand and cure tuberculosis, *M. tuberculosis* infections remain the leading cause of death by an infectious agent. Improvements in socioeconomic conditions and public health interventions led to a decline in tuberculosis cases in industrialized nations the early twentieth century, and the discovery of antibiotics provided therapeutic interventions that vastly improved clinical outcomes for tuberculosis patients. However, the emergence of antibiotic resistant MDR and XDR strains of tuberculosis and the resurgence of tuberculosis cases due to the HIV epidemic in the 1980s returned the disease to the spotlight (Porter and McAdam, 1994). At least a quarter of the world's population is currently infected with active or latent tuberculosis, with over 10 million new infections and 1.2 million deaths from tuberculosis occurring every year. Over 15% of tuberculosis cases occur in the form of extrapulmonary infections that can affect any tissue in the body and are particularly difficult to diagnose and treat (Behr et al., 2018, 2019; WHO, 2019). The challenges facing patients with extrapulmonary infections are indicative of how little we understand this deadly disease, in spite of the long history of research that has been undertaken on the subject. In this review, we will discuss the incidence and diversity of extrapulmonary infections, the role of *M. tuberculosis* dissemination in pathogenesis of by *M. tuberculosis*, and the potential mechanisms of dissemination that *M. tuberculosis* employs to cross the alveolar epithelium and disseminate to secondary sites of infection.

## Extrapulmonary Tuberculosis: Unfamiliar Presentations of a Familiar Disease

The clinical presentations of tuberculosis are well-known both in the medical literature and in popular culture. Active tuberculosis usually presents as a pulmonary infection consisting of a cough lasting longer than a few weeks, often associated with the production of bloody sputum and a myriad of other classic symptoms including chills, fever, weakness, unintentional weight loss, and night sweats. Latent tuberculosis generally does not produce any clinical symptoms, and patients may never know that they have been infected unless reactivation occurs (Esmail et al., 2014). What is less widely known is that in addition to these two extremes, tuberculosis is capable of causing infections in an extremely wide range of tissues and organs. In fact, ~15% of tuberculosis infections worldwide are extrapulmonary infections, that may or may not be accompanied by pulmonary symptoms (WHO, 2019). Extrapulmonary infections pose additional clinical challenges as they do not necessarily mean a patient will test positive for tuberculosis using a sputum smear, the gold standard TB diagnostic (Zurcher et al., 2019). In addition, the presence of *M. tuberculosis* in extrapulmonary locations can result in a wide range of additional symptoms and can pose complications for treatment regimens which already face ongoing challenges in terms of efficacy, compliance, and problematic side effects.

The most common form of extrapulmonary infection in tuberculosis patients is lymphadenitis, most typically infection of the cervical lymph nodes (Peto et al., 2009). In extreme cases, these infections can lead to severe swelling resembling a growth or tumor on the neck. Mycobacterial lymphadenitis was historically referred to as scrofula or the “King's Evil,” as it was widely believed in medieval England and France to be curable through the touch of royalty. This superstition was widespread enough to be referenced by Shakespeare in the play Macbeth (Grzybowski and Allen, 1995). Over time, superstition was gradually replaced by the theory that scrofula was caused by an infectious disease, but it was not until Robert Koch was able to demonstrate the presence of mycobacteria in infected lymph nodes in 1882 that scrofula was understood to be a form of extrapulmonary tuberculosis (Barberis et al., 2017).

Another presentation of extrapulmonary tuberculosis that was once considered to be a separate disease is Pott's Disease, first described by Dr. Percival Pott in 1779. Pott described a palsy of the lower limbs associated with a distinctive curvature of the spine, and an abscess between one or more vertebrae (Dobson, 1972). This condition could be progressive and spread to secondary sites, potentially resulting in paralysis. Today Pott's disease is considered to be synonymous with spinal tuberculosis, a condition so ancient that it has been identified in human mummies in Egypt dating back to 3,400 BC (Taylor et al., 2007). While spinal tuberculosis is the most common form of musculoskeletal tuberculosis, *M. tuberculosis* can also infect any of the bones or joints in the body, commonly described either as articular tuberculosis in which the hips or knee joints are affected, or extraspinal tuberculous osteomyelitis when other localized bone infections occur (Golden and Vikram, 2005).

Similar to infections of the cervical lymph nodes, the second most common form of extrapulmonary tuberculosis is also located in close proximity to the primary site of infection in the lungs. Pleural tuberculosis is an infection of the membranes lining the lungs, often in the form of pleural effusions, or buildup of fluid between the membranes and lung. It was previously thought that pleural effusions were the results of a hypersensitive immune response against pulmonary tuberculosis infections as the pleural fluid was not thought to contain bacteria, but improvements in diagnostic techniques have shown that despite a low bacterial load the pleura is indeed often an active site of extrapulmonary infection (Diacon et al., 2003). Pleural tuberculosis often responds well to treatment and can even resolve spontaneously, but is often associated with later reactivation (Shaw et al., 2018).

Historically, another common presentation of extrapulmonary tuberculosis was infection of the gastrointestinal tract. Interestingly, these infections are more commonly associated with the closely related species *Mycobacterium bovis*, rather than *M. tuberculosis*, most likely due to the consumption of contaminated milk products (de la Rua-Domenech, 2006). *M. bovis*, also known as bovine tuberculosis, is 99.5% genetically identical to *M. tuberculosis* and can be difficult to distinguish from the human pathogen both clinically and immunologically despite the fact that they can be identified as distinct species using PCR and DNA sequencing techniques (Garnier et al., 2003). Despite the genetic and pathogenetic similarity, there is strong species tropism between the two organisms with *M. tuberculosis* being primarily a human pathogen while *M. bovis* naturally infects cattle, buffalo, deer, and even badger populations (Corner et al., 2012). Following the advent of pasteurization of milk products and improved screening methods, the number of gastrointestinal tuberculosis infections decreased dramatically, as this process is generally sufficient to kill mycobacteria (Chalmers, 1945).

A less common but potentially serious form of extrapulmonary tuberculosis is infection of the central nervous system (CNS). This can take the form of tuberculosis meningitis, encephalitis, or as an abscess or tuberculoma (Rock et al., 2008). The origin of infections within the meninges has been hypothesized to be a single focal caseous lesion known as the Rich focus that appears to pre-date the meningitis and is likely to be the source of bacteria that infiltrate the sub-arachnid space (Rich and McCordock, 1933). Tuberculosis infections of the CNS can mimic a number of other serious conditions including meningitis caused by more acute viral, bacterial, or even fungal pathogens or even brain cancer. Taken together with the fact that CNS tuberculosis often presents as non-specific symptoms such as headache, low grade fever, neck stiffness, vomiting, and occasionally cognitive changes, *M. tuberculosis* infections of the CNS can be a diagnostic challenge (Schaller et al., 2019). The prognosis of CNS tuberculosis is particularly poor compared to other forms of tuberculosis, with extremely high mortality rates that are dependent on the stage at which *M. tuberculosis* infection is diagnosed and ensuing complications such as infarctions and hydrocephaly (El Sahly et al., 2007).

The most severe form of extrapulmonary tuberculosis is a systemic infection caused by widespread hematogenous spread of the bacteria. Dissemination throughout the entire body through the bloodstream results in numerous small lesions that can occur on any type. Early physicians considered these ubiquitous lesions to resemble millet seeds, resulting in the term miliary tuberculosis. These lesions can and do occur in every tissue within the body, but are most predominant in organs that are highly vascularized including the lungs, liver, spleen, bone marrow and kidneys (Sharma et al., 2005). In the pre-antibiotic era, miliary tuberculosis was considered to be an infallibly fatal progression of tuberculosis, and as with most forms of extrapulmonary tuberculosis was most commonly seen in young children (Munro, 1889). However, miliary tuberculosis often responds well to modern treatment regimes, and current mortality rates range around 20%, dependent on the age of the patient and other complicating factors (Kim et al., 1990; Lee et al., 2018).

A defining feature of extrapulmonary tuberculosis in every clinical form is the overrepresentation of these infections in vulnerable populations such as children and individuals suffering from malnourishment (Cegielski and McMurray, 2004). In a recent study of pulmonary and extrapulmonary infections in patients in the US between 1988 and 2014, children under 14 years of age were found to be more than twice as likely to have extrapulmonary tuberculosis than pulmonary tuberculosis, despite extrapulmonary infections making up such a small percentage of total cases (Banta et al., 2019). Other risk factors that have been shown to increase the likelihood of extrapulmonary tuberculosis are homelessness, incarceration, and excessive alcohol consumption (Peto et al., 2009). However, the single largest factor influencing the prevalence of extrapulmonary tuberculosis in modern medicine has been the HIV epidemic. As the number of people infected with the HIV virus increased in the 1980's, a concurrent increase in extrapulmonary mycobacterial infections was also observed, often from species of mycobacteria such as *Mycobacterium avium* that rarely cause disease in immunocompetent individuals (ATS CDC, 1987). Although much of this increase is likely due to the overall increase in tuberculosis infections due to HIV co-infection, there is a positive correlation between HIV and extrapulmonary sites of disease (Naing et al., 2013). In a humanized mouse model, HIV infection has been shown to cause a decrease in lung interstitial CD4+ T cells during tuberculosis infections and significant increase in disseminated disease, suggesting a possible mechanism for this association (Corleis et al., 2019).

## *M. tuberculosis* Dissemination: Rare Event, or Mandatory Phase of Infection?

The vast majority of *M. tuberculosis* infections are transmitted through inhaled aerosols, making the lungs the primary site of infection. Therefore, it is widely accepted that dissemination out of the lung is a prerequisite step for most extrapulmonary infections. However, there is also a great deal of evidence that mycobacterial dissemination may be more than just a rare event leading to extrapulmonary tuberculosis. Case studies and pathology of human patients throughout medical history indicate that dissemination may in fact be an essential first step in establishing all active tuberculosis infections, even when these infections present as prototypical pulmonary infections. As early as 1935, Dr. Elizabeth Lincoln noted a trend in the literature away from the previous thinking that disseminated tuberculosis was a rare event inevitably leading to catastrophic outcomes such as miliary tuberculosis, but rather a potential intermediate step of infection (Lincoln, 1935).

The early events following *M*. tuberculosis infection are difficult to follow due to lack of clinical symptoms for most patients at this stage of the disease. Most of our understanding has come from case studies following a recent outbreak, literature from the pre-antibiotic era, and animal studies. Despite the paucity of information, it has long been noted that following initial aerosol infection with *M. tuberculosis* the majority of productive infections occur in a single infection site within one lobe of the lung (Ghon, 1916; Blacklock, 1932). The initial sites of infection are often described as a localized patch of pneumonia, and can occur in any part of the lung (Marais et al., 2004a). Anton Ghon, an Australian pathologist, was one of the first to describe a focus of infection that can occur during this initial infection, lending his name to what is known commonly known as the Ghon's focus. He further described how these initial lesions could progress to include involvement of nearby lymph nodes, creating a cluster of infection known as the Ghon's complex. These primary lesions often calcify during the course of disease, resulting in a distinct pathology. This pattern of infection was once thought to be a hallmark of childhood tuberculosis, but in the modern era where adults in countries with low incidence of tuberculosis are less likely to have been exposed to *M. tuberculosis* it has become evident that this is instead the progression of *M. tuberculosis* infections in immune naive individuals who have not previously developed an immune response against mycobacteria (Loddenkemper et al., 2015).

**Figure 1:**
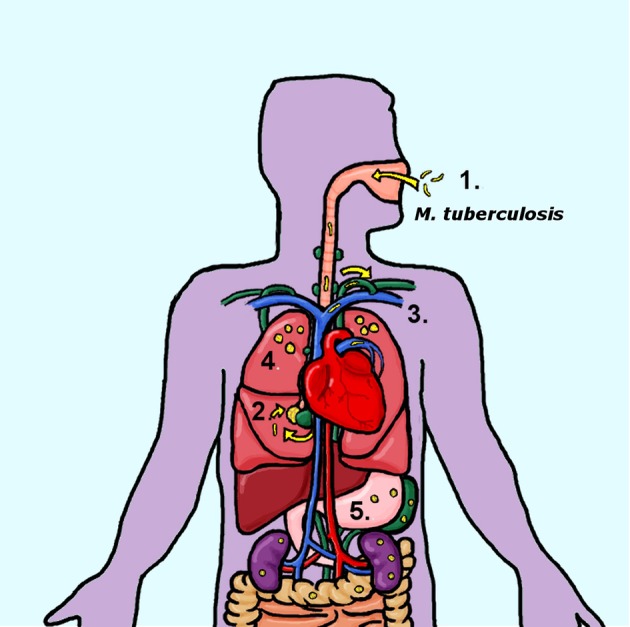
Progression of human *M. tuberculosis* infections (1) Human tuberculosis infections are transmitted through the inhalation of contaminated aerosols. (2) Primary infections are established in the lungs, at a single site that develops into a primary granuloma. These initial sites of infection include involvement of the surrounding lymph nodes, forming a Ghon's complex. (3) Bacteria disseminate out of the lungs and into the lymphatic system, most likely entering the circulatory system through the entry of the thoracic duct into the subclavian vein. (4) Hematogenous reseeding of the lungs results in secondary granulomas located in the apical regions of the lungs and/or the extrapulmonary organs (5).

In contrast to primary tuberculosis infections, secondary or “post-primary” infections that occur either following reactivation or in previously exposed patients often present as numerous foci of infection in the apical and subapical lobes of the lungs (Balasubramanian et al., 1994). This tropism for the apical regions of the lungs has long been noted in patient autopsies and has been a matter of speculation for much of modern medical science. In 1949, Smith and Abernathy reviewed the myriad of theories that could explain the apical localization of post-primary tuberculosis lesions, and added their own hypothesis that *M. tuberculosis* spreads from infected lymph nodes into the lymphatic system, eventually entering the bloodstream through the thoracic duct which empties into the left subclavian vein, entering the heart through the superior vena cava. Assuming minimal mixing of blood from the superior and inferior vena cava, bacilli entering through this route would seed the apical lobes of the lungs by transiting through the bloodstream via the pulmonary artery (Smith and Abernathy, 1949). The idea that *M. tuberculosis* spreads from an initial single lesion to the surrounding lymph nodes, and transits through the lymphatic and circulatory systems to reseed the lungs is supported by earlier observations from pediatric physicians, including Dr. A. Margaret C. Macpherson, who noted that pediatric patients with enlarged lymph nodes near the primary site of infection were more likely to progress to disseminated infections including miliary tuberculosis. Dr. Macpherson hypothesized that this progression was likely due to spread of the bacteria through the lymphatic system and into the bloodstream via the thoracic duct, resulting in hematogenous dissemination (Margaret and Macpherson, 1942). Wallgren made similar observations, hypothesizing that dissemination occurs early, and can result in excretion of bacilli in the urine, though it is seldom possible to culture bacteria from the blood (Wallgren, 1948). Overall, observations in humans, particularly those in children, suggests that hematogenous spread of bacteria occurs co-incident to primary disease (Marais et al., 2004a,b). Since it is reasonable to speculate that hematogenous spread is responsible for secondary lesions in the lung found in most if not all infections (Sweany et al., 1931; Medlar, 1948; Stead, 1989; Balasubramanian et al., 1994), it is likely that bacterial factors also play an important role in this process. This conclusion is supported by the fact that different *M. tuberculosis* strains vary in their ability to cause extrapulmonary infections (Garcia de Viedma et al., 2005). Lymphohematogenous spread remains the most likely path of disease progression for both pulmonary and extrapulmonary infections acquired through the respiratory route (Figure 1).

## *M. tuberculosis* Dissemination in Animal Models

A more detailed understanding of the course of *M. tuberculosis* dissemination must come from animal models. While mice are an obvious model for pathogenesis studies due to their ease of use, low cost, and the availability of tools, reagents and genetic knockouts, it is unclear how closely dissemination in this model mimics what occurs in humans. The morphology of the mycobacterial granulomas differs significantly between mice and humans, suggesting underlying differences in *M, tuberculosis* pathogenesis during mouse infections as compared to human patients (Flynn, 2006). Granulomas are a prototypical characteristic of tuberculosis infections. They are the lesions observed in the lungs and other organs where organized layers of host immune cells surround foci of bacteria, and have long been debated to be either a reservoir of bacteria or a quarantine site regulated by the host (Guirado and Schlesinger, 2013). Whereas, as previously described, the granulomas observed in human infections can be varied in composition including inert calcified lesions and necrotic, caseous granulomas, no necrosis is observable in traditional mouse models (Medlar, 1948; Flynn, 2006). In contrast, the guinea pig model of infection displays two physiologically and immunologically distinct types of granulomas, more closely replicating what is observed in human infections (Ly et al., 2008).

Interestingly, the morphology and cytokine profiles of granulomas observed in the guinea pig model can be traced back to whether they are initial sites of bacterial seeding (primary granulomas), or subsequent secondary granulomas following reseeding of the lungs through lymphohematogenous spread (McMurray, 2003). Infecting animals with very low doses of *M. tuberculosis* results in the formation of a small number of granulomas that eventually become large and necrotic. Similar to what is observed in humans, the primary lesions resulting from the initial infection often become necrotic and calcify. Bacteria disseminate from the primary lesions very early during the course of infection, and within 2 weeks post-infection, bacteria can be found first in the lymph nodes adjacent to the lungs, and then later in the extrapulmonary organs including the spleen (Smith et al., 1970). Approximately 3–5 weeks post-infection, hematogenous dissemination re-seeds the lung, creating secondary lesions that develop primarily in the apical and subapical regions of the lung (Stead, 1989; Balasubramanian et al., 1994; McMurray, 2003). These secondary granulomas are smaller and do not become necrotic or calcify (Ho et al., 1978). Animals that have been vaccinated with BCG develop granulomas that are more similar to secondary granulomas from the onset, suggesting that the differences between these phenotypes is most likely due to the host immune response (Smith et al., 1975). Interestingly, dissemination in guinea pigs occurs in a temporal fashion that is very similar to that observed in mice (Kong et al., 2009). Overall, these observations suggest that the guinea pig model offers the opportunity to examine dissemination in more detail using a highly relevant system to human infections.

Rabbit models of tuberculosis have been useful for studying tuberculosis due to the characterization of both resistant and sensitive rabbit models. Lurie's sensitive rabbit model showed disease similar to that demonstrated in guinea pigs including extrapulmonary dissemination and distinct primary and secondary granulomas (Lurie, 1941). However, the majority of rabbit experiments were performed with *M. bovis* as rabbits do not develop severe disease or extrapulmonary infections from *M*. tuberculosis (Nedeltchev et al., 2009). Perhaps the ultimate model for studying *M. tuberculosis* dissemination and extrapulmonary spread is non-human primates who closely resemble human patients in terms of their susceptibility and immune response to *M. tuberculosis*. Similar to human infections, *M. tuberculosis* infections of cynomolgus macaques results in extrapulmonary infections in only a subset of animals. This allows more accurate modeling, but also makes studying extrapulmonary infections more complicated as they do not occur in every experimental animal. Interestingly, treating macaques with TNF neutralizing agents resulted in drastically increased extrapulmonary dissemination and the development of disseminated disease within 8 weeks post-infection (Lin et al., 2010). Moreover, similar to patterns of human infections in the pre-antibiotic era, macaques that do not show extrapulmonary infections in other organs still harbor persistent infections within their lymph nodes, suggesting that the lymphatic dissemination model is correct (Ganchua et al., 2018). The application of tools such as PET scans and genetic labeling of bacterial in non-human primate infections suggests that this model is likely to shed deeper insight into the mechanisms of *M. tuberculosis* dissemination in the future (Martin et al., 2017).

## Breaching the Barrier: A question of mechanism

From what we have learned over the past century from both observations of human patients and experimental animal models, the likelihood of *M. tuberculosis* initiating infection from a single site and disseminating through the lymphatic and/or circulatory system is incredibly high. However, relatively little is understood about the molecular mechanisms of dissemination. Based on the correlation between susceptibility to severe disease and the frequency of extrapulmonary infections in various animal models, it can be assumed that the host immune response to infection plays a major role. This conclusion is further substantiated by the link between immunodeficiency and extrapulmonary tuberculosis in humans. However, there is also evidence that *M. tuberculosis* is not a passive player in this process and that bacterial virulence factors actively promote dissemination. There is evidence that *M. tuberculosis* strains from different phylogenetic lineages show different rates of extrapulmonary disease, and clinical isolates from extrapulmonary infections cause a greater degree of disseminated disease in animal models (Hernandez Pando et al., 2010; Be et al., 2011; Click et al., 2012). Furthermore, there is evidence that *M. tuberculosis* actively induces angiogenesis to promote dissemination through the formation of new blood vessels (Oehlers et al., 2015; Polena et al., 2016).

**Figure 2:**
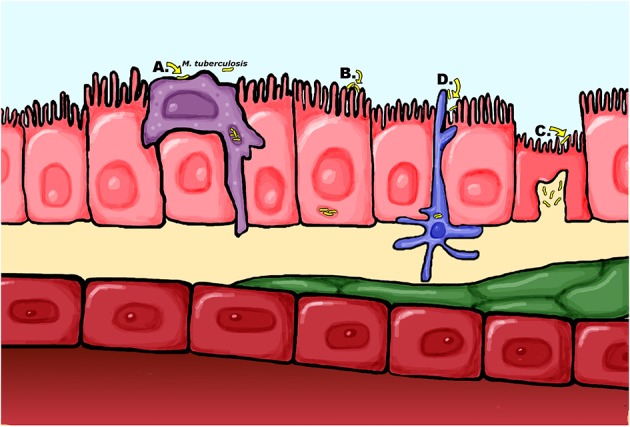
Proposed mechanisms of *M. tuberculosis* dissemination across the airway epithelia **(A)** The “trojan horse” model of dissemination where *M. tuberculosis* is carried across the epithelial barrier within infected macrophages. **(B)** *M. tuberculosis* is capable of directly infecting epithelial cells, which could result in bacteria translocating across the barrier through the epithelial cells or inducing cell death to cause a breach in the barrier **(C)** Passage across the epithelium may occur in specialized M cells which actively translocate antigens to the interstitium for presentation to antigen presenting cells **(D)** Alternatively, dendritic cells sampling antigens in the alveoli may traffic live mycobacteria to the lymph nodes.

Of particular interest from a mechanistic viewpoint is the essential first step of extrapulmonary dissemination, the egress of *M. tuberculosis* out of the lung. For mycobacteria to gain access to interstitial tissues, it would first need to cross the epithelial barrier of the lung, circumventing the primary purpose of barrier epithelia. A number of models have been proposed regarding how non-motile bacteria could breach the lung epithelium, which we will discuss in more detail (Figure 2). One hypothesis is that as *M. tuberculosis* preferentially infect alveolar macrophages in high numbers, the bacteria could be transiting within these macrophages as they cross into and out of the lymphatic and circulatory systems. Another hypothesis is that *M. tuberculosis* directly infects the epithelial cells composing the barrier of the lung and is able to either translocate across these cells without disrupting the epithelium, or causes a breach in the monolayer by inducing cell death (Russell, 2001). Alternatively, dissemination could involve a role from a less ubiquitous cell type within the lung, dendritic cells, which are known to sample antigens from the alveoli and present them within the lymph nodes (Humphreys et al., 2006). Interestingly, there are genetic and phenotypic evidence available in the literature for all three hypotheses regarding routes of dissemination, possibly suggesting that in reality dissemination may occur by a combination of several or all of these pathways.

## Macrophage Migration: The Trojan Horse theory

The “trojan horse” theory of dissemination hypothesized that *M. tuberculosis* traffics within alveolar macrophages (AM) across the airway epithelium. This is an attractive theory, because it is well-established that AMs are one of the first and most numerous types cells to become infected with tuberculosis in both humans and animal models (Berthrong, 1970; Wagner, 1975; Srivastava et al., 2014). *M. tuberculosis* can survive and replicate within AMs, and the ability of this cell type to cross into and out of the circulatory and lymphatic systems is well-characterized. Moreover, this mechanism has been demonstrated in other bacterial pathogens, suggesting it could be a conserved mechanism for bacterial dissemination (Vazquez-Torres et al., 1999). However, the role of macrophages in *M. tuberculosis* dissemination and extrapulmonary spread is still not completely understood and knockouts in animal models are not usually specific enough to allow definitive demonstration of their role. Possibly, tissue-specific or lineage-specific knockouts in mice could allow careful analysis of their role in future studies.

Some evidence for trafficking of mycobacteria within macrophages comes from a zebrafish model using the related pathogen *Mycobacterium marinum*. Zebrafish provide a useful model to study to progression of mycobacterial infections because the natural transparency of the larvae allows the progress of infection to be followed in real time (Davis et al., 2002). Using this model, Davis and Ramakrishnan showed direct evidence of macrophage recruitment to *M. marinum* granulomas and subsequent migration of infected macrophages to new tissues (Davis and Ramakrishnan, 2009). The zebrafish model has also resulted in the identification and characterization of a number of bacterial factors that appear to play a role in dissemination of mycobacteria, including Zinc metalloprotease-1 (z*mp1*) *and* the regulatory gene *whiB6*, as well as the host factor CXC chemokine receptor 3 (CXCR3) (Torraca et al., 2015; Chen et al., 2016; Vemula et al., 2016). Interestingly, *M. marinum* mutants lacking the ESX-1 secretion locus, which is essential for full virulence in *M. tuberculosis* and *M. bovis* infections, were found to show decreased granuloma formation but increased dissemination within macrophages to remote tissues, suggesting an important role for this locus in dissemination.

While the zebrafish model provides evidence for the trojan horse theory of mycobacterial dissemination, this model cannot provide proof of dissemination across the alveolar epithelium or address the roles of the lymphatic system and adaptive immune system in dissemination. In addition, while *M. marinum* is a natural pathogen of fish and thus an excellent model for mycobacterial pathogenesis, it is divergent enough from *M. tuberculosis* that it will be important to confirm these findings in the actual human pathogen. A virulence factor that has been studied in relation to dissemination in both *M. marinum* and *M. tuberculosis* using a mammalian model is the virulence locus *mel2* that affects growth in activated macrophages as well as entry into host cells and dissemination (Subbian et al., 2007; Cirillo et al., 2009). The *mel2* locus affects susceptibility to reactive oxygen species (ROS) and this role may be the basis for effects on intracellular growth, but it is unclear whether this role is responsible for the effects on dissemination, since dissemination of a *mel2* mutant remains defective in phox^−/−^ and iNOS^−/−^ mice (Subbian et al., 2007; Cirillo et al., 2009).

Direct evidence for the trojan horse model in mammals was reported by Cohen et al. (2018). This study utilized an intratracheal antibody labeling assay to show that alveolar macrophages (AMs) infected with mCherry-labeled *M. tuberculosis* migrate from the lumen of the alveoli, where they typically reside, to the interstitium (Cohen et al., 2018). This was an exciting and interesting observation, as AMs have previously been described as sessile cells that remain closely associated with the epithelium even when stimulated (Westphalen et al., 2014). The authors investigated the genetics of this process, determining that migration is dependent on the *M. tuberculosis* virulence factor ESX-1, corroborating observations in the zebrafish model. Furthermore, using gene knockout mice, bone marrow chimeras, and adoptive transfer experiments, they demonstrated a role for the host IL-1R signaling pathway in dissemination through a mechanism that is dependent on non-hematopoietic cells, most likely epithelial cells. Not only is this a potentially important host-side mechanism in *M. tuberculosis* dissemination, but it suggests that mycobacterial dissemination may not be solely dependent on exploitation of a single host cell type, but rather the entire alveolar environment.

## Epithelial Cell Infection: The Direct Approach

Perhaps the most direct strategy to breach the alveolar barrier is to directly infect the cells that make up the barrier. Once inside an epithelial cell, bacteria could translocate across the cells or induce apoptosis or necrosis, causing a break in the epithelium due to cell death. Mycobacteria has been known to be capable of infecting HeLa cells since the 1950's (Bloch, 1956; Shepard, 1957), and was shown to be capable of growth and replication in human lung epithelial cells over 20 years ago (Bermudez and Goodman, 1996; Mehta et al., 2006). The same year, the first *M. tuberculosis* adhesin, the heparin-binding hemagglutinin HbhA was identified and shown to be involved in adhesion of *M. tuberculosis* to epithelial cells (Menozzi et al., 2006). Infection of epithelial cells has also been shown to induce chemokine expression suggesting that this cell type could also play an important role in the immune response against *M. tuberculosis* (Lin et al., 1998; Wickremasinghe et al., 1999). Analysis of infected cells present in human sputum and in bronchiolar lavage samples shows that epithelial cells become infected with *M. tuberculosis* in human patients and in fact are one of the most commonly infected cell types after macrophages and neutrophils (Eum et al., 2010).

Transit of *M. tuberculosis* across epithelial cells has been demonstrated *in vitro* using polarized bilayers of epithelial and endothelial cells to recreate the airway barriers of the human lung in culture. In these three-dimensional models, epithelial cells and endothelial cells are cultured and allowed to polarize on opposite sides of a permeable transwell membrane. *M. tuberculosis* is then added to the apical chamber, and the basal chamber is monitored for bacteria that are able to translocate across the cell bilayer, showing that the bacteria is able to migrate across the epithelial and endothelial cell barriers (Birkness et al., 1999; Bermudez et al., 2002) In addition, Pethe et al. published a study in 2001 on the previously characterized adhesin HbhA that demonstrated a clear role for this gene in dissemination through interactions with epithelial cells. They showed that deletion of *hbhA* from either *M. tuberculosis* or the human vaccine strain *Mycobacterium bovis* BCG had no effect on the ability of the bacteria to infect or grow within the lung, but significantly impaired the ability to disseminate to the spleen following intranasal infection (Pethe et al., 2001). Moreover, they showed that *hbhA* mutants had no phenotype whatsoever in J774 macrophage cells, but were impaired in their ability to infect A549 human lung epithelial cells, consistent with the role of this gene in cell adhesion. Overall, these studies provide evidence for a direct role for epithelial cells in dissemination.

The role of *hbhA* in dissemination across epithelial cells has since been confirmed *in vitro* using the polarized bilayer models described above (Ryndak et al., 2016). However, these experiments do not address whether dissemination is due to translocation across epithelial cells, or cell death. Purified recombinant HbhA binds to the surface of polarized epithelial cells, induces actin reorganization, and can be internalized into cytoplasmic vacuoles via endocytosis. However, HbhA does not disrupt the integrity of cellular tight junctions or affect the permeability of epithelial cell monolayers (Menozzi et al., 2006). In contrast, infection of polarized monolayers with live bacteria does affect the isoelectric properties of epithelial cell monolayers, possibly by inducing TNFα expression, suggesting that there could be additional HbhA-independent mechanisms of barrier disruption (Zhang et al., 1997).

Another possible route of passage across epithelial cells could be through specialized epithelial cells known as microfold cells, or M cells. M cells are part of the epithelial barrier in mucosa-associated lymphoid tissues (MALT) including the gut and some parts of the respiratory system. Although M cells form tight junctions with other epithelial cells and are part of the epithelial barrier, they play an active role in taking up antigens and delivering them across the epithelia to antigen-presenting cells (Neutra et al., 1996). Due to this unique ability, M cells have been shown to be exploited by a large number of bacterial intestinal pathogens to invade deeper tissues (Owen et al., 1986; Grutzkau et al., 1990; Jones et al., 1994). The first suggestion that mycobacteria could also be using M cells as a route across the epithelia came in 1986, via demonstration of uptake of the *M. bovis* BCG vaccine strain by M cells in a rabbit ileum ligated loop model (Fujimura, 1986). A similar role for pulmonary M cells was later demonstrated in the guinea pig model using virulent *M. tuberculosis* (Teitelbaum et al., 1999).

Additional evidence for the role of M cells in mycobacterial dissemination was provided more recently using the mouse model to demonstrate that depletion of M cells decreases dissemination to the cervical lymph nodes, and an *in vitro* model using Caco-2 epithelial cells to show increased translocation when M cells are co-cultured in the monolayers (Nair et al., 2016). Taken together, these studies suggest that M cells are capable of translocating *M. tuberculosis* and likely play a role in dissemination. The only downside to this model of dissemination is that the prevalence of M cells in the human lung epithelium is unclear. Both nasal-associated lymphoid tissues (NALT) and bronchus-associated lymphoid tissues (BALT) are present in rodent models, but very little is known about these tissues in healthy human adults. They have been described as being primarily present in childhood and subsequently receding, perhaps inducible in response to infection or inflammation or only sparsely present (Bienenstock and McDermott, 2005). Therefore, it remains somewhat unclear what role M cells play in *M. tuberculosis* dissemination in humans, and it is likely that this is not the sole route that mycobacteria may disseminate through.

## Dendritic Cell Infiltration: Opportunistic Hitchhikers

Another candidate cell population suggested to play a role in *M. tuberculosis* dissemination are dendritic cells (Humphreys et al., 2006). These cells are particularly attractive candidates due to the established role of dendritic cells in active transport of antigens to the lymph nodes. Therefore, dendritic cells could provide a potential route of dissemination out of the primary site of infection for *M. tuberculosis* as they transport bacteria to the lymph nodes for presentation to immune cells. This hypothesis is supported by data showing that infection of dendritic cells by BCG can occur within 48 h following intranasal infection of BALB/c mice, a timepoint that is relevant to early dissemination out of primary granulomas and formation of the Ghon's complex (Reljic et al., 2005). Moreover, infected inflammatory dendritic cells (iDCs) defined as CD11c+CD11b+Ly6C+ cells are capable of moving into and out of acute and chronic lesions induced by BCG in a CD11c-eYFP dendritic cell reporter mouse strain. Importantly, iDCs in dendritic cell reporter mice infected with BCG also migrate to peripheral sites at a much higher rate than in uninfected mice (Schreiber et al., 2011). Harding et al. also show that iDCs in the same reporter strain are recruited to *M. tuberculosis* granuloma-like lesions, after which they are found outside of the lesions interacting with populations of P25 cells and forming new regions of granulomatous inflammation (Harding et al., 2015).

Although these experiments establish a link between dendritic cells and dissemination, they have all been performed in mouse models that do not form structurally similar primary and secondary granulomas to those observed in humans. Many of these experiments also used the model organism *M. bovis* BCG, rather than virulent *M. tuberculosis*, so there remains a need to confirm the results of these novel experiments using *M. tuberculosis*. Interestingly, both human and mouse dendritic cell migration decreases across an epithelial barrier toward lymphatic chemokines following infection with the attenuated *M. tuberculosis* strain mc^2^7000 or BCG (Roberts and Robinson, 2014; Harding et al., 2015). However, a computational model extrapolated from a data set consisting of blood and lung samples of non-human primates infected with the Erdman strain of *M. tuberculosis* also predicted an essential role for dendritic cells in dissemination, suggesting that dissemination within dendritic cells can occur in human tuberculosis (Marino and Kirschner, 2016).

Dendritic cells have also been described as playing a “trojan horse” role in transiting other respiratory pathogens to the lymph nodes, setting a precedent that could extend to *M. tuberculosis*. Cleret et al. observed transit of fluorescent-labeled *Bacillus anthracis* spores to the thoracic lymph nodes in GFP-labeled dendritic cells (Cleret et al., 2007). Subsequent studies suggest similar roles for DCs in trafficking *Streptococcus pneumoniae* and *Francisella tularensis* from initial infection sites in the lungs to the lymphatic system as well as roles for DCs in systemic dissemination of *Burkholderia pseudomallei* and *Salmonella typhimurium* (Bar-Haim et al., 2008; Rosendahl et al., 2013; Williams et al., 2014; Carden et al., 2017). Taken together, these studies suggest that dendritic cells play a prominent role in bacterial dissemination and that this may be a conserved mechanism across bacterial species.

## Discussion

Extrapulmonary tuberculosis accounts for a relatively small percentage of human tuberculosis cases in immunocompetent adults. However, the clinical impact of extrapulmonary tuberculosis is larger than this statistic may suggest as extrapulmonary infections are some of the most difficult to diagnose and treat. The gold standard for diagnosis of tuberculosis in many countries with the highest tuberculosis burdens remains sputum smear microscopy, but patients with extrapulmonary infections do not necessarily have bacteria in their sputum. Other assays that can be used to test for exposure to mycobacterial antigens, such as the tuberculin skin test and the QuantiFERON blood test are limited due to cross-reactivity with the BCG vaccine or environmental mycobacteria, respectively, and thus cannot be used to diagnose clinical tuberculosis. In 2010, GeneXpert was introduced and recommended by the WHO for pulmonary infections, but although this has the potential to address diagnostic challenges the test requires sophisticated and expensive equipment that is not always available in the places it is most needed and remains a sputum-based test (Walzl et al., 2018). Overall, this means that diagnosis strategies are lacking for extrapulmonary infections. The wide range of sites and symptoms associated with extrapulmonary tuberculosis means that it can masquerade as a number of different diseases and syndromes, such that tuberculosis may not even be suspected and tested for, delaying the time before appropriate treatment can be provided. Even when extrapulmonary tuberculosis is diagnosed in a timely manner, the recommended treatment regimen are primarily designed against pulmonary tuberculosis, and may or may not be effective depending on the presentation of extrapulmonary disease.

Extrapulmonary tuberculosis is associated with particularly high morbidity and mortality. This may be due to the fact that extrapulmonary forms of the disease often occur in some of the most vulnerable patients including young children and immunocompromised individuals. However, while they are no longer the death sentence that they once were, certain forms of extrapulmonary tuberculosis, particularly infection of the central nervous system such as meningitis and miliary tuberculosis, have very poor clinical outcomes. Diagnosis and morbidity/mortality are closely related in extrapulmonary tuberculosis, as early detection of infections can drastically improve the likelihood of the disease responding to treatment.

As most tuberculosis infections are contracted through the inhalation of aerosols containing *M. tuberculosis*, extrapulmonary infections occur through dissemination of the bacteria out of the lung into the surrounding lymphatic tissue, and subsequent distribution throughout the circulatory system. Secondary pulmonary granulomas are formed through reseeding of the lungs through the bloodstream. Therefore, understanding dissemination has broad implications for tuberculosis treatment and prevention. If these early steps can be blocked through vaccination or early intervention, it is conceivable that not only could extrapulmonary infections be prevented, but that reseeding the lungs could be blocked. This hypothesis is supported by the success of therapeutics designed to target the first known *M. tuberculosis* dissemination factor, *hbhA*. Immunization with purified recombinant HbhA protects mice from infection with *M. tuberculosis*, reducing the bacterial burden in both the lungs and extrapulmonary organs (Parra et al., 2004; Schepers et al., 2015). Boosting with this antigen also improves the efficacy of the BCG vaccine, suggesting a combined regimen has the potential to protect against dissemination (Guerrero et al., 2010). HbhA has also been investigated as a potential diagnostic antigen and biomarker (De Maio et al., 2019), suggesting that work in this area can be applied in a number of novel ways.

It is not unreasonable to think that identification and characterization of additional dissemination factors could lead to the development of equally successful vaccines and therapeutics. However, since the identification of HbhA, research in this area has resulted in only a few new candidates being identified. Further investigation into identifying mycobacterial dissemination factors is needed. Identification of a comprehensive set of *M. tuberculosis* dissemination and extrapulmonary spread factors could improve our understanding of the molecular mechanisms involved, which will need to be confirmed and further evaluated in both small animal models and *in vitro* models. More sophisticated tracking of dissemination *in vivo* using modern imaging techniques could allow analysis of the longstanding belief that dissemination occurs via the lymphatic and circulatory systems. Moreover, further investigations into the interactions of *M. tuberculosis* with their host cells could help us better understand the mechanisms that the bacteria use to breach the alveolar barrier and cross into the lymphatic and/or circulatory systems.

Guidance on the future of *M. tuberculosis* dissemination research may come from the progress of research in other bacterial pathogens. The route through which enteric pathogens such as *Salmonella typhimurium* disseminate across the intestinal endothelial barrier to infect other tissues was once a hotly debated topic in bacteriology. Closely paralleling the current state of understanding of *M. tuberculosis* dissemination, the two major schools of thought were that *S. typhimurium* was either directly invading the epithelium though Microfold (M) cells, or hitching a ride within migrating macrophages. This debate was eventually addressed through experiments using bacterial genetics to address each hypothesis. First, *S. typhimurium* was demonstrated to exploit M cells by using bacterial adhesins to invade and colonize Peyer's Patches (Galan and Curtiss, 1989; Lee et al., 1992; Jones et al., 1994). A subsequent study showed that if all proposed epithelial cell adhesins were deleted from *S. typhimurium*, the resultant triple knockout mutant was still able to disseminate within a mouse model, though at a reduced level. Moreover, if the triple mutant was used to infect CD18 KO mice that lack a surface antigen expressed by macrophages and dendritic cells that dissemination to the liver and spleen was greatly reduced compared to wild-type mice (Garcia de Viedma et al., 2005). From these combined studies, it can be concluded that neither of these proposed mechanisms are mutually exclusive, and that *S. typhimurium* likely exploits both potential dissemination routes. To bring a similar sense of conclusion to the *M. tuberculosis* field, it will be necessary to perform similarly careful genetic studies that clarify the role of each proposed pathway in a relevant *in vivo* model such as non-human primates or the guinea pig model of infection. Using the history of enteric pathogen dissemination as a lesson, it seems likely that none of the proposed theories are mutually exclusive and that future evidence may reveal that mycobacteria are capable of utilizing more than one strategy to disseminate and establish extrapulmonary infections.

## Author Contributions

MM wrote the article and prepared the figures. JC provided the concept and critical review.

### Conflict of Interest

The authors declare that the research was conducted in the absence of any commercial or financial relationships that could be construed as a potential conflict of interest.
